# Serial recall in spatial acoustic environments: irrelevant sound effect and spatial source alternations

**DOI:** 10.1038/s41598-025-18592-9

**Published:** 2025-09-12

**Authors:** Cosima A. Ermert, Manuj Yadav, John E. Marsh, Sabine J. Schlittmeier, Torsten W. Kuhlen, Janina Fels

**Affiliations:** 1 Institute for Hearing Technology and Acoustics, RWTH Aachen University, Aachen, Germany; 2 Human Factors Laboratory, School of Psychology and Humanities, University of Lancashire, Preston, UK; 3 Department of Health, Learning and Technology, Luleå University of Technology, Luleå, Sweden; 4 Faculty of Society & Design, Bond University, Gold Coast, Queensland, Australia; 5 Work and Engineering Psychology, RWTH Aachen University, Aachen, Germany; 6 Visual Computing Institute, RWTH Aachen University, Aachen, Germany

**Keywords:** Auditory verbal serial recall, Short-term memory, Spatial audio, Irrelevant sound effect, Spatial source alternation effect, Psychology, Engineering

## Abstract

This study investigated serial recall performance in a complex acoustic scene that included spatialised background sounds and location changes within the target sequence to reflect real-life challenges. The focus is on two effects: the irrelevant sound effect (ISE) and the spatial-source alternation effect (SSAE). Both represent impairment in short-term memory performance of to-be-remembered items: the ISE due to irrelevant background sounds, and the SSAE due to location changes within the target sequence. Although distinct, these effects typically occur together in real-world settings, e.g., listening to multiple speakers in noise, but have not been investigated together yet. Building on the theoretical frameworks of these two effects, this study combines principles from both the irrelevant sound effect (ISE) and the spatial-source alternation effect (SSAE) as a step towards enhancing acoustic complexity in established cognitive tasks. Experiment 1 examined auditory-verbal serial recall using spatially alternating target digits presented at a typical rate (1 item/1 s), with either meaningful or meaningless background speech. Results showed an ISE, with meaningful speech causing greater disruption, but no SSAE - possibly due to either the presentation rate or the spatialised audio scene. To further clarify this, Experiment 2 was conducted with a faster presentation rate (1 item/350 ms) consistent with a previous study, and more spatial target locations (monotic, $\pm 10^{\circ }, \pm 45^{\circ }, \pm 60^{\circ }, \pm 90^{\circ }$). An SSAE was revealed for all locations. These findings suggest that the SSAE may mainly be modulated by the presentation rate—given the spatial separation is audible and only emergent at rapid location changes—questioning its applicability to naturalistic listening scenarios. As an attempt to bridge the gap between controlled laboratory settings and more complex listening tasks, these findings help explain how cognitive systems manage competing demands in real-world auditory environments, such as separating speech streams in noise.

## Introduction

Auditory distractions are pervasive and can significantly disrupt our ability to process and remember information^1–4^, often without us fully realising it^5,6^. When following multi-talker conversations in noise, listeners have to regularly refocus their attention on the active speaker and suppress potential background noise, including competing conversations, while simultaneously processing the spoken content of interest. Within cognitive psychology, auditory distraction has been widely studied and has provided key insights into the mechanisms of attention, memory, and perception^7,8^. However, much of this research has focused on static auditory settings^9,10^, where the sound sources remain stationary and the effects of dynamic location changes that are typical in everyday settings—refocussing attention across active speakers—are not fully considered. Consequently, much less is known about the effect of changes in the location of sound sources on memory performance of spoken content. Understanding these dynamics is essential both from a theoretical point of view but also for designing environments and systems that minimise distraction and optimise performance.

A well-established paradigm in this context is the serial recall test, in which participants have to remember and recall a sequence of verbal items, e.g., digits or words, in the presentation order^11^. Serial memory of aurally presented items is often taken as a proxy for the ability to listen to speech and maintain the content for subsequent recall^12,13^. However, the restricted dynamism of the auditory scenes in terms of the location and direction of the sound sources typical to studies of this paradigm^12,14–19^ limits the generalisation of findings to everyday communication settings, such as listening to conversations between spatially separated talkers under competing noise.

This study investigates how location switches in target speech and spatialised background noise impact short-term memory performance of aurally presented verbal information, as measured within the serial recall paradigm. The influence of background noise and location switches in target speech on memory performance can be modeled with the ISE and SSAE, respectively. The ISE^9^ describes how task-irrelevant background sounds disrupt the recall of to-be-remembered items. The SSAE^20,21^ (sometimes alternating-ear-effect) describes how location changes during the presentation of to-be-remembered items impede performance compared to static presentation. While both the ISE and the SSAE represent disruptions in short-term memory performance for auditory material and are both usually examined using verbal serial recall tasks, they involve distinct mechanisms and contexts. The ISE arises from task-irrelevant auditory stimuli such as background speech, whereas the SSAE concerns task-relevant auditory stimuli, specifically focusing on spatial changes in the presentation of the to-be-remembered items. The theoretical concepts and findings underlying ISE and SSAE will be explained in more detail in the following.

The ISE^9^ has proven to be a robust finding, enhancing our theoretical grasp of auditory cognition^8,9,22,23^ and providing practical implications for the acoustic optimisation of learning and working environments like classrooms and open-plan offices, where cognitively demanding tasks have to be performed in the presence of noise^24–26^. According to the duplex-mechanism account^2^, the ISE is characterised by two primary mechanisms: variations in the spectral content of the task-irrelevant sound over time (changing-state effect) and its capacity to capture attention. Changing-state sounds (e.g., sequences of syllables or letters like “A-B-C-D”, words, running speech, backward speech), as opposed to more steady-state sounds (e.g., noise, pure tones, or repeated letters like “A-A-A-A”), contain order information arising from spectro-temporal changes in the auditory signal which competes with the order information in the target sequence of the serial recall, disrupting serial short-term memory performance^27^. The attentional capture mechanism, by contrast, occurs when a salient feature in the background sound, such as meaningful speech, alerting words like one’s own name, or an unexpected deviant^2^ (e.g., a unique item in a sequence like “A-A-B-A-A”), momentarily diverts the listener’s attention away from the primary task^2^.

Most studies investigating the ISE use serial recall of visually presented verbal items (e.g., words, letters, digits). Compared to visual item presentation, research using auditory presentation is limited. This can partly be attributed to the potential of target items being masked by the background noise when presented auditorily. It is hence crucial to ensure that background noise does not affect the perception or intelligibility of the presented items, as any performance decrement could otherwise be attributed to inaudibility/masking rather than memory-related effects^14^. Moreover, auditory presentation of target items is highly relevant to many everyday scenarios (e.g., communication settings), wherein both the target and noise occur in the auditory modality. Therefore, the effects of auditory presentation warrant deeper exploration in studies of the ISE, in particular the effects of spatialised background noise and target items. Such investigations can bridge the gap between controlled experimental conditions and the complexity of real-world environments.

In the few studies where the ISE has been investigated using serial recall tasks with auditory presentation, several types of task-irrelevant sounds have been used. These have included sequences of letters or syllables^14–16^, intelligible but unrelated words^17,18^, running speech in the listener’s native^16^ or non-native language^19^, and music^12^. Most of these studies used non-spatial audio reproductions, such as headphones or mono/stereo loudspeaker setups. This differs from realistic scenarios, where sounds—both background and target—are typically accompanied by spatial information. Such information may include differences in intensity and time of arrival of a sound across the ears and direction-dependent changes in the spectral content of the sound, which are evaluated by higher auditory processing to analyse and navigate complex auditory scenes^28^. Spatial audio reproduction of background sounds has been employed in very few visual-verbal serial recall studies (e.g., with spatial loudspeaker setups^29–33^ or virtual acoustic scenes presented via headphones^34^) and even fewer auditory verbal serial recall studies (e.g., with virtual acoustic background scenes and non-spatialised target presented via headphones^24^). However, there are no previous studies that have included realistic spatial representation of background sounds and target items.

Another challenge in everyday listening environments is spatial changes in target signals, since target speakers are often spatially separated, requiring listeners to dynamically allocate attention across spatial locations^10^. This provides a strong motivation to investigate the effect of spatial changes in a target sequence on short-term memory performance, which is studied in the context of the SSAE^20,21^. In this phenomenon, a spatial illusion has traditionally been created by presenting target items alternately to each ear via headphones (i.e., monotically), leading them to be perceived as originating from two distinct spatial locations (although still being localised in the head). While this reproduction technique has limitations in terms of spatial plausibility, it serves as an important initial step towards exploring the influence of spatial target signals in verbal serial recall.

Two prominent theoretical accounts have been proposed to explain the mechanisms underlying the SSAE: the item-encoding account and the perceptual-motor account. The item-encoding account argues that switching attention between spatial locations^35^ (or voices^36–40^) during encoding—especially at fast presentation rates—places strain on cognitive resources, impairing recall. In contrast, the perceptual-motor account^21,41^ emphasises a mismatch between how listeners perceive and group incoming information and the demands of the recall task. For instance, if digits alternate between left and right locations (e.g., left-right-left-right), listeners may group them into two separate perceptual streams based on location. However, the task still requires a single, linear reproduction of the sequence in its original order. This creates a “mapping conflict”: the perceived structure of the input does not match the required structure of the output. The motor component of this account refers not to overt motor execution, but to the challenge of translating two competing perceptual streams into one ordered response. Both accounts predict disruption under spatial alternation, but they differ in where the disruption arises - at encoding or during output planning.

While our approach draws heavily from research on auditory distraction and verbal serial recall^2^, the current study also intersects with broader findings in auditory cognition. For instance, spatial separation of competing sources has long been known to aid target identification in complex auditory scenes^42–44^, and changes in spatial and acoustic cues can influence auditory streaming and attentional allocation^45,46^. However, few studies have explored these dynamics in the context of short-term memory paradigms. By combining principles from auditory distraction research with more naturalistic spatial manipulations, our study aims to combine spatialised background and target sound sources within the serial recall paradigm to imitate more complex and realistic auditory scenes in cognitive research. The present paper reports two experiments: Experiment 1 examines serial recall performance in a simplified conversational scene with spatial target and background sources with background speech varying in semantic content as well as location changes, applying principles from both the ISE and SSAE. As a follow-up study designed to assess the mechanisms underlying the SSAE, Experiment 2 explored the emergence of the SSAE without background sounds, and with target sounds presented at faster rates and from a larger number of spatial locations.

## Experiment 1

This experiment aims to investigate verbal serial recall performance with spatial auditory target and background sources, as well as location changes within a digit sequence; reflecting an analytic description of a natural conversation. Based on this, we designed a spatial setup of target and background sound sources to mimic a multi-talker conversation in noise, where two talkers are the target talkers and two separate talkers emit background speech. Three target location patterns were evaluated based on Experiment 3 by Hughes et al.^21^ in the context of SSAE: no location changes, regularly alternating changes, and random changes between two fixed target locations. Instead of the monotic playback employed by Hughes et al.^21^ (i.e., presenting the sequence to one ear at a time), sound is spatialised using a head-related transfer function (HRTF). Monotic playback can be disadvantageous due to in-head localisation where both target and noise stimuli seem to originate from “inside the head” with ambiguous directional separation. In contrast, simulating virtual spatial sound sources using HRTFs increases spatial resolution and enables externalisation for listeners. This enhances sound source localisation and the ability to distinguish between competing auditory signals^28^ and is an established procedure in cognitive research^10,24,25^. The three target patterns were combined with three background noise types based on the ISE study by Yadav et al.^24^: silence, semantically meaningful and intelligible speech, and speech that did not convey meaning to the listener.

The voice delivering the target sequences did not change with the location, as would occur in a real conversation. While having distinct voices for each target location more closely resembles real-life scenarios, it has been shown that voice changes within a target sequence disrupt verbal serial recall of spoken distractors (talker-variability effect)^20^. Hence, if we had introduced distinct voices for each target location, the effect of alternating voices and locations could not have been disentangled^41^. Since a primary aim is to investigate the effect of spatialised target and background sounds within the serial recall, this trade-off in terms of realism was considered acceptable.

### Method

#### Participants

$N = 27$ participants (16 female, 11 male), aged 21–39 years (*M* = 26.34, *SD* = 4.47) were recruited for the experiment via posters displayed at the authors’ institutes and via mailing lists. All participants had German as their first language (self-reported) with normal hearing and (corrected-to) normal vision. Normal hearing was defined, according to the WHO criteria^47^, as a maximum of 25 dBHL (decibels Hearing Level) between 250 Hz and 8 kHz. Hearing was examined using an AURITEC Ear 3.0 audiometer and Sennheiser HDA300 headphones, employing pulsed pure tone ascending audiometry. Vision, aided if necessary, was tested with a Snellen chart (20/30)^48^. The experiment procedure was pre-approved by the Ethics Committee at the Medical Faculty of RWTH Aachen University (EK396-19). Written informed consent was obtained from all participants before the experiment. Each participant received 10 Euro as compensation for their time and involvement. The study was conducted in accordance to the rules of conduct stated in the Declaration of Helsinki.

#### Paradigm

Short-term memory performance was examined with the verbal serial recall using auditory digit presentation. Each trial consisted of four phases: countdown, presentation, retention, and serial reconstruction. In the countdown phase, three rectangles decreasing in size were displayed sequentially on a computer monitor. Each rectangle was followed by a 500 ms pause, resulting in a total countdown time of 1.5 s. In the presentation phase, a sequence of spoken digits (1–9) was played through headphones at a rate of one digit per second. Each spoken digit had a duration of 600 ms, followed by a 400 ms interstimulus interval, resulting in a total presentation duration of 9 s. During this phase, the computer monitor displayed only a play button, with no visual representation of the digits. The digit sequences were designed such that each digit (1–9) appeared exactly once within a sequence. Furthermore, only two consecutive steps of one were permitted (e.g., 1-2-3 or 3-2-1), avoiding longer consecutive progressions (e.g., 1-2-3-4 or 4-3-2-1)^49^. Additionally, each digit appeared in each serial location approximately equally often across trials. The retention phase lasted 3 s during which the participants retained the presented sequence. In the reconstruction phase, the digits 1–9 were displayed in randomised order in a $3\times 3$ grid on the computer screen (Calibri, 80 pt $\hat{=}$ 1.4 ms). Participants were instructed to click the digits in the exact order they were presented. Once a digit was clicked, it disappeared from the grid and corrections were not permitted. After completing the sequence, the next trial began automatically. There was no time limit for completing the reconstruction phase.

The chosen presentation rate of one item per second is well established in serial recall studies^12,14–19^, while slower presentation rates can also be found^50,51^. In their study on the SSAE, Hughes et al.^21^ used a presentation rate of 1 digit per 350 ms, to facilitate spatial stream segregation. Conversational alternations in naturalistic settings occur at a much slower rate than once every 350 ms^52,53^. Thus, a presentation rate of one digit per second was selected instead.

#### Audio reproduction

**Fig. 1:**
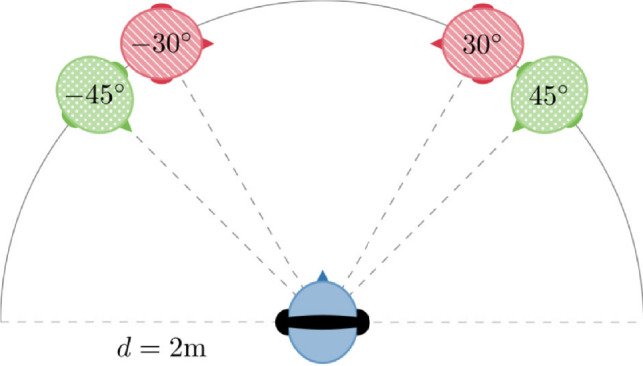
Setup in Experiment 1. Target sources () were placed virtually at $\pm 45^{\circ }$, noise sources () at $\pm 30^{\circ }$ on the horizontal plane at a distance of $d =2~\hbox {m}$ from the receiver () and played back via headphones.

A simulated auditory scene replicating a conversational environment was created using Virtual Acoustics^54^. Binaural spatial signals were generated by convolving the auditory stimuli with a generic HRTF of an artificial head^55^. Two target sources, playing back digit sequences for the auditory serial recall task, were located at $\pm 45^{\circ }$ on the horizontal plane (see Fig. 1) in a virtual free field without room acoustics.

The receiver (participant) and the two target sources (at $\pm 45^{\circ }$) formed an equilateral triangle with two additional noise sources (at $\pm 30^{\circ }$) on the horizontal plane, resulting in a $15^{\circ }$ angular offset between each target and noise source pair. Based on the minimum audible angle, i.e., the smallest angular separation at which two sound sources are perceived as arising from two separate locations^56^, for non-individual HRTFs^57^, this spatial arrangement provides sufficient separation for the participant to perceive distinct target and noise sources. In this study, all virtual sound sources were located 2 m away from the receiver (i.e., listener location), a distance considered comfortable for interactions with unfamiliar individuals^58^. To simulate a conversational scenario, the target sound sources were oriented toward the receiver, while the noise sources were oriented toward each other. This arrangement mimicked a conversation between the noise sources.

Playback of all sounds was delivered through Sennheiser HD650 headphones with individualised headphone equalisation^59^, using an RME Fireface UC soundcard. The receiver’s location was tracked using Optitrack and Motive software (v1.10.0), enabling dynamic reproduction to counteract head movements. This approach not only resembles real-life listening conditions but also enhances localisation accuracy compared to static reproductions^60^.

#### Stimuli

For the target stimuli, recordings of German digit words spoken by a female voice (female b)^61^ were used. For the *noise*, three types of stimuli were created: *silence*, *meaningful speech*, and *pseudo-speech*.

The *meaningful speech* condition consisted of intelligible German sentences taken from the AuViST database^62^, which contains 34 family stories narrated in 10 sentences each. Recordings are available for both female and male speakers. Sentences with digit words were excluded. The remaining sentences were presented in a randomised order^63^. While coherent narratives might have elicited higher ecological validity, this approach ensured stimulus control and variability, while preserving acoustic features of running speech, such as prosody and temporal dynamics. One background source used a female voice, and the other a male voice, balanced across participants to control for voice-related effects.

The *pseudo-speech* was designed to convey no meaning to the listener and be unintelligible (in contrast to *meaningful speech*) while maintaining the same temporal-spectral structure as the *meaningful speech*, to control for differences in energetic masking. For that matter, pseudo-speech was generated by cutting the sentences from the *meaningful speech* into syllables and randomly rearranging them to create unintelligible sequences for both female and male voices in Audacity (v3.1.3). A 10 ms cross-fading was applied between syllables to avoid artifacts.

Both noise sources emitted their signal simultaneously and continuously, without breaks between sentences. Although this approach differs from a realistic background conversation, it was chosen to avoid spatial-temporal changes that might exceed those caused by the temporal structure of the speech. Additionally, the continuous emission ensured a symmetrical design, maintaining consistent spatial release from masking benefits for the target source.

All stimuli were calibrated using the HEAD acoustics HMS III artificial head. Target stimuli were calibrated to 60 dB(A) and the background stimuli were adjusted to 57 dB(A), measured as the power sum across both ears at the receiver’s location. Due to the spatial separation of the target and background sound sources, an additional spatial release from masking benefit^64,65^ of up to 6 dB can be expected^66^.

#### Target location patterns

Three *patterns* were implemented for the target location changes: no location changes (*single*), regularly alternating changes (*alt-reg*), and random irregular alternations (*alt-irr*) between two fixed target locations. These were adapted from Experiment 3 in Hughes et al.^21^, with the difference being that their study used eight digits, whereas this experiment used nine digits. In the *single* condition, all digits in the target sequence were played back from either the left or right side, with the side balanced across trials. In the *alt-reg* condition, the target source location alternated regularly between left and right for each digit (e.g., LRLRLRLRL), with the starting direction (left or right) balanced across trials. In the *alt-irr* condition, the target source location alternated in an irregular, pseudo-random manner. A maximum of two consecutive digits could be presented from the same side, and the total number of digits presented from each side was balanced (five from one side, four from the other). The side emitting the majority of digits was also balanced across trials.

#### Procedure

Each participant completed the experiment in an individual session inside a sound-insulated hearing booth. The session began with audiometric testing, a Snellen vision test, and headphone equalisation measurement. Only participants who passed these screening procedures proceeded to the main experiment. Participants were seated on a wooden chair (seat height: 0.44 m) located 1.0 m away from an LG Flatron L1710B, with the monitor’s center at a height of 1.1 m. Written instructions were displayed on the monitor. Participants were explicitly instructed not to vocalise the digits, use their fingers as aids, or rely on other movements for support. To familiarise themselves with the procedure, participants first completed a practice block, consisting of six silent trials in the single condition. Three practice trials were presented from $+45^{\circ }$ and three from $-45^{\circ }$, balanced across trials. Following this, participants listened to the *meaningful speech* and *pseudo-speech* and completed one practice trial each, with the order of conditions balanced. To ensure intelligibility, participants confirmed they could understand all the digits before proceeding to the main experiment. The main experiment comprised nine blocks of eight trials each, for a total of 72 trials. Each block included a unique combination of the variables *pattern* (*single*, *alt-reg*, *alt-irr*) and *noise* (*silence*, *meaningful speech*, *pseudo-speech*). In blocks with background noise, the noise signal played continuously without breaks throughout the block. The order of block presentations was counterbalanced across participants using a Latin square design. Participants were allowed to take voluntary breaks of no fixed length between blocks. Additionally, a mandatory 2 min break was administered after the fifth block. The total duration of the session, including the screening process, was approximately 60 min. The experiment was implemented using MATLAB (v2023a), with the ITA-toolbox^67^.

#### Data analysis

The statistical analyses were conducted using R (version 4.2.2). The fixed effect, i.e., dependent variable, was the recall accuracy in the task, defined as the proportion of digits recalled at the correct serial location. The independent variables were the *noise* conditions (*silence*, *meaningful speech*, *pseudo-speech*), and the target *pattern* (*single*, *alt-reg*, *alt-irr*). Separate Bayesian generalised mixed-effects models were created using the R package brms (version 2.18)^68^. In each model, the random effects were incorporated as independently varying intercepts and independently varying and uncorrelated slopes across the serial locations, and the independent variables (*noise* and *pattern*) for the participants. A zero-one inflated Beta distribution with a logit link function was used, suitable for proportion data bounded between 0 and 1; several relevant distributions (e.g., Gaussian) were used for comparison.

Models were built incrementally, starting with an intercept-only baseline model, and progressively adding random effects, independent variables and their interactions. Weakly informative priors (in the logit scale) for the distributions per factor and their interactions were specified, and sampling included 16,000 iterations (four independent chains of 5000 samples each, with the first 1000 discarded as warm-up). Model performance was compared using leave-one-out (LOO) cross-validation, evaluating differences in expected log pointwise predictive density and the standard error of the expected log pointwise predictive density of the model^69^. Posterior predictive checks performed per model confirmed the models adequately captured the observed data distributions. Orthogonal contrasts were conducted across the factor levels, with results summarised using medians and 95% credible intervals (CIs), where statistically robust effects refer to the 95% CI, not spanning zero. credible intervals (CIs), 95% CI, calculated using the highest density interval of the posterior probability distribution, provides the interval within which 95% of the posterior probability distribution lies. We use the proportion of the 95% CI inside the region of 225 practical equivalence (ROPE) to determine whether an effect is meaningful/significant. ROPE is the range signifying an effect of negligible magnitude and is conceptually similar to the null hypothesis in frequentist statistics. The range for the ROPE was specified as $\pm 0.1 \cdot SD_{\mathrm {dependent~variable}}$^70^, and calculated using the R package bayestestR^71^.

### Results

**Fig. 2:**
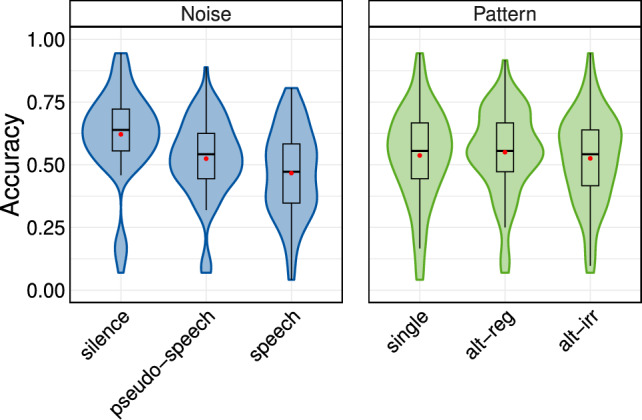
Results of Experiment 1. The accuracy distribution (averaged over the serial location) is displayed for the independent variables *noise* and *pattern*. The mean is indicated with a red dot ().

The distributions of data are illustrated in Fig. 2. Based on the LOO comparisons across models, the model including only *noise* performed best, outperforming other models that also included *pattern* or an interaction between the predictor variables. This indicates that *pattern* was not a robust predictor of recall accuracy in the serial recall task. As summarised in Table 1, recall accuracy was significantly higher in the *silence* condition compared to the average accuracy in the *speech* conditions (*meaningful speech* and *pseudo-speech*). Furthermore, recall accuracy was significantly lower in the *meaningful speech* condition than in the *pseudo-speech* condition. Although the contrasts across the *pattern* conditions were not statistically significant, they are presented here for reference and potential use in future studies.

**Table 1:** Orthogonal contrasts for recall accuracy across the *noise* conditions (top two rows; significant effects), and the *pattern* conditions (bottom two rows; non-significant effects).

Contrast	Median	95% CI	% in ROPE
*silence* – *speech*	0.13	[0.11, 0.16]	0
*meaningful speech* – *pseudo-speech*	−0.05	[−0.07, −0.03]	0
*single* – *alternating*	0.003	[−0.01, 0.02]	100
*alt-reg* – *alt-irr*	0.02	[0.0, 0.04]	80.5

CI, Bayesian credible interval; ROPE, region of practical equivalence

### Discussion

Two main findings can be derived from the experiment, as shown in Table 1: both *meaningful speech* and *pseudo-speech* as background noise decreased recall performance (ISE), with a stronger impact of *meaningful speech*. Secondly, the *pattern* of location switches in the target signal did not affect recall performance (SSAE), and the factors *noise* and *pattern* did not interact.

The ISE for meaningful background speech was stronger than for pseudo-speech. Similar effects have been demonstrated for serial recall with visually^72,73^ and aurally^24^ presented digits. One possible explanation of this effect is a difference in masking between the signals, potentially challenging the encoding of the items. Masking occurs if the audibility of a target signal is reduced when presented concurrently with an irrelevant sound and would therefore affect the perception or encoding of the items within the serial recall task. A distinction is made here between informational and energetic masking. Energetic masking is observed if the target and irrelevant signal contain energy in the same frequency range, causing parts of both signals to be inaudible, while informational masking is a higher-level cognitive process that occurs when both signals are comprehensible, making it harder to separate the target from the noise^42,64,65^. While it can be assumed that *pseudo-speech* primarily elicited energetic masking, in the *meaningful speech* condition, informational masking was present in addition^74^. It is, however, unlikely that the performance difference only stems from this masking difference, possibly impeding performance in the encoding phase of the serial recall, since intelligibility in all conditions was ensured and this “intelligibility” effect can be found in visual serial recall tasks as well^72,73^. The duplex-mechanism account^2^ justifies different effects of the noise signals in the recall phase: both signals elicited a changing-state effect, but the meaningful speech additionally had the capacity to capture attention. This finding underscores that the content’s semantic meaning has a disruptive impact on recall of auditory verbal items, as noted in previous studies^24^.

Splitting up the target sentence across two spatial locations did not impede memory performance. This implies that listeners were able to treat the spatially separated speech as a single coherent stream in the sequence reconstruction—likely because the presentation rate and consistent vocal characteristics may have facilitated re-grouping the items despite location changes. This stands in contrast to scenarios where either rapid presentation or multiple distinct talkers introduce greater perceptual load, making it more difficult to group items and potentially leading to reduced recall fidelity^75^. The traditional finding of the SSAE is often examined under different conditions with faster presentation rates (350 ms per digit^21^ vs. 1 s per digit in this study) and without spatial audio (monotic^21^ vs. spatialised in this study). From a theoretical point of view, both of these changes could have contributed to the absence of the SSAE. Both the item-encoding hypothesis^35^ and the perceptual-motor account^21,41^ can be drawn upon to explain the absence of the SSAE due to the presentation rate. From the perspective of the item-encoding hypothesis, the slower rate may have reduced demands on encoding spatial changes, allowing sufficient time for processing and attenuating potential disruptions. The perceptual-motor account suggests that slower rates might not create perceptual groupings strong enough to conflict with the planning and sequencing of a response; a stronger memory effect for increased rates can thus be predicted from both accounts. Although the dependency of SSAE on presentation rates has not been directly tested, slow location changes may not induce sufficient spatial disruption to elicit an SSAE. A rate dependency has, e.g., been found for alternating voices instead of alternating locations within a target sequence (talker-variability effect^38^). To explain the present findings comprehensively, the influence of rate on the SSAE should be investigated further.

The extent to which the spatial audio reproduction contributed to the absence of the SSAE is unclear since the SSAE has not been investigated together with spatial sound sources (background or target) so far. Contradictory predictions can be derived from the item-encoding hypotheses and the perceptual-motor account with regard to the effect of spatial sound and, consequently, the angle of target source separation on the SSAE. The item-encoding account attributes the SSAE to a cost in attention switching between the target source positions and/or between the ears as input channels in a more strongly-worded formulation of the account^35^. The cost of attention switching, which has been shown to be dependent on the switching angle^76^, results in an angle-dependent SSAE. If switching the ear as the input channel is the driving factor, the SSAE would only emerge for monotic presentation, as spatialised audio presents the stimulus to both ears albeit with a level difference. In contrast, the perceptual-motor account would predict that perceivable spatial changes in the target sequence, even at minimal angles, would maintain SSAE effects due to “streaming-by-location”, where sequences are processed as originating from distinct spatial locations^21^.

While both factors—presentation rate and spatial audio reproduction—may have contributed to the absence of the SSAE in this study, their roles in increasing the auditory realism in serial recall tasks are distinct. As previously discussed, spatial audio is essential for naturalistic listening environments. If the use of spatial stimuli had led to the absence of the SSAE, this would have strong implications for designing experimental scenarios that better approximate real-world listening. However, listeners in everyday situations are unlikely to process alternations as rapid as every 350 ms. Consequently, if the SSAE is only observed at such fast presentation rates, its relevance for understanding or simulating typical real-life auditory experiences would be limited. Thus, to explain the effects of this study comprehensively, there is a need to investigate the SSAE with a faster rate and under varying spatial conditions, including larger variations in the extent of location changes, which is studied further in Experiment 2.

## Experiment 2

In Experiment 1, a presentation rate of one item per second was chosen. Typically, faster rates are used to investigate the sequential integration of items into a perceptual stream^77^. To ensure that the SSAE is limited to high presentation rates and to further examine how spatial audio presentation contributed to the absence of the SSAE, Experiment 2 replicates the presentation rate of 1/350 ms from Hughes et al.^21^ and extends the range of aural presentation angles. No background noise was included in this experiment to enable us to compare multiple target source locations within one experiment. The locations included the monotic condition (playing the raw digit stimulus one ear at a time), which was also employed by Hughes et al.^21^. The spatial auralisation closest to the monotic reproduction places virtual sound sources at $\pm 90^{\circ }$ on the horizontal plane. In addition to this maximal angular change, we included a smaller separation of $20^{\circ }$ ($\pm 10^{\circ }$ relative to frontal location), which is a small but audible change in location based on the expected minimum audible angle of the present system. Additionally, we chose $\pm 45^{\circ }$ for comparability with Experiment 1, and $\pm 60^{\circ }$ to expand the grid of angular sampling.

Different hypotheses can be formed for this experiment, depending on the underlying cognitive processes. The item-encoding account explains the SSAE as arising from the temporal cost associated with shifting attention, such as the left and right ears as distinct input channels^35^. When stimuli are presented at a fast rate, these attentional shifts may not keep pace with the item presentation rate. However, spatial, in contrast to monotic, presentation involves auditory information being available to both ears (with interaural level differences), so the ears no longer function as independent channels but information from both ears is evaluated. Consequently, the SSAE should only be observed in the monotic condition, where input channels remain distinct. However, it has been shown that the time required to shift auditory spatial attention between two target talkers depends on the magnitude of the angular change^76^. Thus, in a more general interpretation of the item-encoding account one could also expect the SSAE to vary with the angular distance between successive items based on the predictions of the item-encoding account.

Alternatively, if the SSAE is driven by streaming-by-location as proposed in the perceptual-motor account, then any perceived change in sound location—regardless of the size of the shift—could segment the target sequence into separate streams. While for each individual stream the order information is stored, this segmentation would disrupt the reconstruction of the original order from the different streams, potentially affecting both monotic and spatial conditions.

### Method

#### Participants

*N* = 30 adults (16 female, 14 male), aged 19–44 years ($M = 27.83$, $SD = 6.05$) participated in the experiment. Recruitment, screening procedures, ethics approval, written consent, and compensation were consistent with Experiment 1.

#### Paradigm

The same paradigm as in Experiment 1 was used, with the exception that the item presentation rate was adjusted to match that of Hughes et al.^21^. Digits were presented for 250 ms with an interstimulus interval of 100 ms, resulting in a total item duration of 350 ms per digit. Consequently, a sequence duration was reduced from 9 (Experiment 1) to 3.15 s.

#### Audio reproduction

**Fig. 3:**
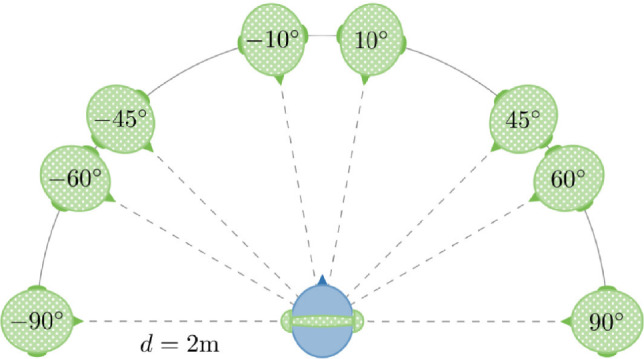
Setup in Experiment 2. Target sequences () were reproduced spatially or *monotic* via headphones. Spatial target sources were placed virtually at $\pm 10^{\circ }$, $\pm 45^{\circ }$, $\pm 60^{\circ }$, $\pm 90^{\circ }$ on the horizontal plane at a distance of $d=2~\hbox {m}$ from the receiver ().

The methods of audio reproduction remained identical to those in Experiment 1, with the addition of more sound source locations in Experiment 2, as illustrated in Fig. 3. Five *angles* were examined: $\pm 10^{\circ }, \pm 45^{\circ },\pm 60^{\circ }, \pm 90^{\circ }$, and *monotic* playback (lacking spatial information). The same dynamic playback and headphone equalisation methods were applied as in Experiment 1.

#### Stimuli

New stimuli were required to accommodate the faster presentation rate. Initially, the stimuli from Experiment 1 were shortened to 250 ms using Audacity’s (v3.1.3) time-compression algorithm, which preserved the original spectral distribution. However, this strong post-processing introduced substantial distortion. As a result, new recordings were made with a female speaker in a sound-isolated booth using a Rode NT5 microphone and a Zoom H6 Handy Recorder. A distance of approximately 1 m between the speaker’s mouth and the microphone was maintained as in the stimuli from Experiment 1^61^. The speaker was instructed to articulate quickly and clearly. Time-compressed recordings from Experiment 1 were used as a reference during the recording. Each digit was recorded multiple times in mixed orders to ensure consistent articulation. Post-processing was conducted in Audacity (v3.1.3), where recordings were cut and normalised following the European Broadcasting Union R128 standard, targeting an average loudness of –23 LUFS relative to Full Scale^78^. The final recordings were between 201 ms and 346 ms in duration (*M* = 271.34 ms, *SD* = 37.22 ms). These were further adjusted to 250 ms using Audacity’s time-compression algorithm. Stimuli were calibrated to 60 dB(A) using the same procedure as in Experiment 1.

#### Target location patterns

To minimise participant fatigue and to focus on key contrasts between *single* and *alt-reg* conditions, the *alt-irr* condition from Experiment 1 was omitted. Only two *patterns* were employed: *single* and *alt-reg*.

#### Procedure

The equipment and general procedures mirrored those in Experiment 1. Adjustments were made to the training block and the main block conditions. The training block consisted of six trials with diotic sound presentation (i.e., identical stimuli presented to both ears without spatial information). The main experiment included 10 blocks, each corresponding to a unique combination of the variables *pattern* (*single*, *alt-reg*) and *angle* ($\pm 10^{\circ }, \pm 45^{\circ }, \pm 60^{\circ }, \pm 90^{\circ }$, *monotic*). The background noise as an additional factor were omitted. A balanced latin square design was used to control the order of conditions. Participants could take breaks between blocks, with a mandatory 2 min break after the fifth block. The total duration of the experiment was approximately 45 min.

#### Data analysis

For the two independent variables, *pattern* (*single*, *alt-reg*) and *angle* ($\pm 10^{\circ }, \pm 45^{\circ }, \pm 60^{\circ }, \pm 90^{\circ }$, *monotic*), the statistical analyses included the same steps as described in the data analysis section for Experiment 1, except for using a Gaussian distribution as the family for the accuracy, and using random slopes only for the serial position. The latter two choices were based on the LOO comparisons, where the Gaussian distribution provided a substantially better fit than the zero-one-inflated Beta distribution used in Experiment 1, and the model with random slopes for the independent variables did not improve the fit compared to the model with random slopes just for the serial position.

### Results

**Fig. 4:**
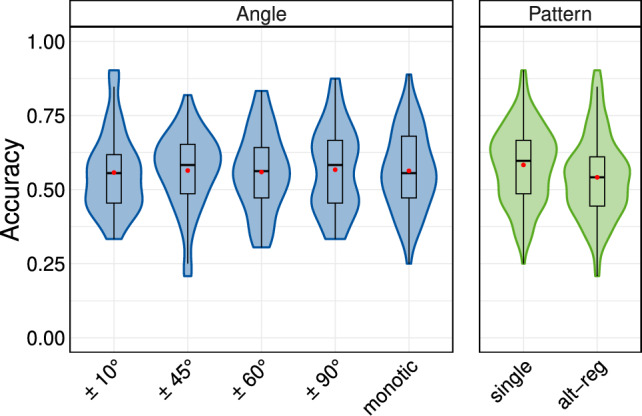
Results of Experiment 2. The accuracy distribution (averaged over the serial position) is displayed for the independent variables angle and pattern. The mean is indicated with a red dot ().

The distribution of results is visualised in Fig. 4. Based on the LOO comparisons, the model including only pattern outperformed models that included angle or an interaction between the factors. The difference in accuracy between the *single* condition (all digits presented to one side) and the *alt-reg* condition (alternating locations with each digit) was statistically significant (Table 2). However, the angle did not result in statistically significant differences in accuracy.

**Table 2:** Pairwise contrasts for recall accuracy across the *pattern* (top row; significant effect) and *angle* conditions (rows 2–10; non-significant effects).

Contrast	Median	95% CI	% in ROPE
*single* – *alt-reg*	0.04	[0.03, 0.05]	0
*monotic*–10$^{\circ }$	5.89e-03	[−0.01, 0.02]	100
*monotic*–45$^{\circ }$	−2.19e-03	[−0.02, 0.02]	100
*monotic*–60$^{\circ }$	4.40e-03	[−0.01, 0.02]	100
*monotic*–90$^{\circ }$	−3.43e-03	[−0.02, 0.02]	100
10$^{\circ }$–45$^{\circ }$	−7.96e-03	[−0.03, 0.01]	100
10$^{\circ }$ –60$^{\circ }$	−1.42e-03	[−0.02, 0.02]	100
10$^{\circ }$ –90$^{\circ }$	−9.34e-03	[−0.03, 0.01]	100
45$^{\circ }$ –60$^{\circ }$	6.48e-03	[−0.01, 0.02]	100
45$^{\circ }$–90$^{\circ }$	−1.34e-03	[−0.02, 0.02]	100
60$^{\circ }$–90$^{\circ }$	−7.83e-03	[−0.03, 0.01]	100

CI, Bayesian credible interval; ROPE, region of practical equivalence

### Discussion

The analysis revealed that the *angle* ($\pm 10^{\circ }, \pm 45^{\circ }, \pm 60^{\circ }, \pm 90^{\circ }$, *monotic*) resulted in better performance for sequences where all digits were presented from one side (*single*) compared to sequences where the presentation direction alternated with each digit (*alt-reg*). This effect did not vary substantially across the levels of *angle*. The fact that SSAE was observed only in Experiment 2, where a faster presentation rate (1 item / 350 ms) was employed and not in Experiment 1, which used a slower rate (1 item / 1 s), demonstrates rate dependency. A similar rate dependency has been detected for the varying voices between digits^38^. The emergence of SSAE at faster rates was predicted by both the perceptual-motor and item-encoding accounts. Based on this rate dependency, no significant decrease in performance due to shifts in source locations is to be expected in realistic conversational situations—relevant to the broader aim of this paper—as location changes typically occur at slower rates in everyday conversations (generally< 1/s^53^).

The lack of differences across angles aligns with the streaming-by-location explanation^21^, where changes in angle are perceptually grouped into a single spatial stream when rates are fast. This finding challenges the item-encoding account’s prediction that different angular separations change encoding demands and modulate SSAE effects; a strictly angle-dependent version of the item-encoding account would predict that variations in angular separations influence attentional switching demands and therefore differentially affect performance. However, more general formulations of the item-encoding framework, in which any reliable spatial alternation taxes encoding due to changes in co-verbal information, could still explain the observed lack of variation across angles^36^. In summary, since the SSAE was not significantly affected by presentation angle, it seems that once the prerequisites for SSAE are met, any perceivable source separation can be assumed to be sufficient to trigger streaming-by-location. Importantly, an SSAE can occur with both spatial audio reproduction and monotic reproduction at fast rates, reinforceing the importance of perceptual organisation.

## Limitations

While the main aim of Experiment 1 was to increase the realism of the auditory presentation in the serial recall paradigm, it still represented a very controlled setting with multiple differences to real-world scenarios. Further improvements could include diversifying the target voices, incorporating room acoustic influences to increase acoustic complexity, and providing talker visualisations which would aid following the turn-taking. Furthermore, the realism of the target as well as background speech was limited. Background speech could be further improved by including coherent narratives and conversational dynamics between the background talkers, both of which were decided against in the present study to ensure control. The serial recall paradigm itself also limits the realism of the presented stimulus material (digits with pauses in between utterances). Other paradigms, e.g., with running speech^10^, and partially overlapping utterances could possibly reflect naturalistic conversations better and lead to different findings. Still, in order to systematically translate established findings from memory research to more naturalistic contexts, a gradual increase in realism within an established and controlled task such as serial recall was considered an appropriate and effective approach. As for Experiment 2, an SSAE was observed for fast presentation rates. While a joint analysis of the ISE and SSAE at this faster rate would be warranted, particularly to establish a precise rate threshold at which the SSAE emerges, such an analysis was deemed beyond the scope of the current study’s objectives of focusing on improving the realism of acoustic environments within the serial recall paradigm.

## Conclusion

Taking inspiration from everyday conversations, which take place in the presence of background noise and where target speakers are often spatially separated, this study investigated serial recall performance applying principles from both the ISE and SSAE. Two experiments were conducted using serial recall with spatialised auditory target items, focusing on background speech (Experiment 1) and spatial alternation of the target speech stream (Experiments 1 and 2).

Our findings highlight two primary insights. First, irrelevant meaningful speech disrupts short-term memory performance more strongly than pseudo-speech, suggesting that semantic processing captures attention beyond changing-state characteristics in ways pseudo-speech does not. Second, spatial alternation within the target sequence was only shown to impair recall when items are presented at a sufficiently fast rate (here: 1 item per 350 ms), irrespective of how large the angular separation between alternating locations is.

From a practical standpoint, these results imply that in typical conversational settings—where speakers generally alternate turns at slower speeds—the impact of frequent spatial shifts on memory may be minimal. Although rapid switching between different locations can disrupt recall in a controlled lab task, such rapid alternations are uncommon in the real world. Background speech that carries semantic information reduces memory performance, underscoring the disruptive potential of meaningful speech regardless of spatial configuration.

On a theoretical level, our data suggest that once listeners detect a spatial alternation, the separation angle does not influence the degree of disruption at high item presentation rates. However, everyday conversations are rarely carried out at these fast rates. To better understand the temporal dynamics of auditory short-term memory and enhance predictions about memory performance in real conversational settings, future studies should investigate the precise cut-off rate for the emergence of the SSAE and whether further irregular alternation patterns can isolate the cognitive mechanisms behind the effect. Further, the realism of the experimental setting could be increased by incorporating factors like continuous speech, alternating voices, continuous spatial movement, room acoustics, and visual co-verbal cues. Collectively, our findings suggest that spatial separation of talkers is unlikely to affect short-term memory performance under normal presentation rates.

## Data Availability

The datasets used and/or analysed during the current study available from the corresponding author on reasonable request.
